# Mifepristone inhibits non-small cell lung carcinoma cellular escape from DNA damaging cisplatin

**DOI:** 10.1186/s12935-018-0683-z

**Published:** 2018-11-15

**Authors:** Heather E. Kapperman, Alicia A. Goyeneche, Carlos M. Telleria

**Affiliations:** 1 Experimental Pathology Unit, Department of Pathology, Faculty of Medicine, McGill University, 3775 University Street, Montreal, QC H3A 2B4 Canada; 2 Division of Basic Biomedical Sciences, Sanford School of Medicine, The University of South Dakota, Vermillion, SD 57069 USA; 3 Present Address: Eisenhower Army Medical Center, Ft. Gordon, GA USA

**Keywords:** Lung cancer, Mifepristone, Cisplatin, Cell repopulation, Chemotherapy, Clonogenic survival

## Abstract

**Background:**

Lung cancer is the leading cause of cancer deaths in the world. The major histopathological subtype of lung cancer is non-small cell lung cancer (NSCLC). Platinum-based therapy is the standard of care for patients with advanced stage NSCLC. However, even with treatment, most patients will die of this disease within 5 years and most of these deaths are due to recurrence. One strategy to inhibit recurrence is to use cytostatic compounds following courses of lethal chemotherapy. We have shown in various cancer cell types that mifepristone (MF), an anti-progestin/anti-glucocorticoid, is a powerful cytostatic anti-cancer agent. Thus, in this work we tested the hypothesis that MF should be efficacious in inducing cytostasis and preventing repopulation of NSCLC following cisplatin (CDDP) therapy.

**Methods:**

We established an in vitro approach wherein human NSCLC cells with different genetic backgrounds and sensitivities to CDDP (A549 and H23) were exposed to rounds of lethal concentrations of CDDP for 1 h followed or not by MF monotherapy. Every 2 days, cell number, cell viability, and colony-forming ability of viable cells were studied.

**Results:**

CDDP killed the majority of cells, yet there were remnant cells escaping CDDP lethality and repopulating the culture, as evidenced by the improved clonogenic survival of viable cells. In contrast, when cells exposed to CDDP where further treated with MF following CDDP removal, their number and clonogenic capacity were reduced drastically.

**Conclusion:**

This study reports that there is repopulation of NSCLC cells following a lethal concentration of CDDP monotherapy, that NSCLC cells are sensitive to the growth inhibition properties of MF, and that MF abrogates the repopulation of NSCLC cells following CDDP therapy. Our study supports further evaluating MF as an adjuvant therapy for NSCLC.

## Background

Lung cancer is the leading cause of cancer death worldwide [1]. The most common histopathological subtype accounting for about 85% cases is non-small cell lung cancer (NSCLC) [2]. Despite during the past two decades there have been advances in early detection and in the development of targeted therapies that improved prognosis following standard of care with platinating agents, the overall survival rates from NSCLC are still very low; the high mortality of this disease is consequence of the presence of metastases at the time of diagnosis in most patients [3]. Consequently, new drugs and combination therapies are desperately needed to improve the outcome of this fatal cancer.

Mifepristone (MF) is a well-known synthetic steroid that has been approved to be used as antiprogestin in reproductive medicine when blockage of progesterone action is needed, and as antiglucocorticoid to prevent the hyperglycemia associated to Cushing’s disease [4]. However, several studies have shown MF to be useful controlling cell growth as well. For instance, MF was able to control disease expansion in patients with meningioma [5], and block the growth of cancer cells of gastric [6], breast [7], prostate [8], and ovarian [9, 10] origin. Our laboratory has previously shown that MF blocks cancer cell growth by arresting the cells at the G1/S transition via blockage of cyclin dependent kinase 2, and, consequently, inhibition of DNA synthesis [11]. We have also shown that MF is able to potentiate the chemotherapeutic effects of cytotoxic drugs such as cisplatin and paclitaxel [12, 13], as well as the toxicity of proteasome inhibitors by causing aggravation of the stress of the endoplasmic reticulum [14]. Furthermore, we demonstrated that neither relative chemosensitivity nor genetic background were obstacles for MF to display its anti-cancer effects [15, 16].

In cancer carriers, MF may work, at least in part, by blocking cellular repopulation following platinum-based chemotherapy [12, 13]. Repopulation of cancer cells is defined as the continuous proliferation of tumor cells that survive fractionated radiotherapy or chemotherapy [17]. It takes place without changes in chemosensitivity, limits the efficacy of anti-cancer treatment approaches, and ameliorates overall tumor reduction contributing to clinical recurrence [18, 19]. Further, it has been demonstrated that in certain cases the repopulation of cancer cells following chemotherapy or radiotherapy is accelerated, denoting the devastating consequences of the process for a patient. The repopulation phenomenon has been attributed to cells that escape initial treatment by surviving in an environment where most of other tumoral cells die. Several reasons have been postulated to explain this phenomenon, including the concept that cancer stem cells are the ones capable of resisting initial treatment and repopulate a tumor, that a rare percentage of cancer cells undergo, instead of cell death, transitory senescence before regaining proliferation capacity, that a percentage of cancer cells, while not dying from treatment, undergo a process of dormancy from which they awake and regrowth when the conditions around the tumor improve, or, that therapy causes the formation of giant hyperploid cells, some of which have the capacity to reverse into a near diploid stage with the capacity to repopulate the tumor [20, 21].

In this work we used MF against NSCLC which is currently treated with platinum-based chemotherapy. We hypothesized that the genetic background of NSCLC that makes the cells responsive to cisplatin would be similar to that of other cancers sensitive to cisplatin and responsive to MF, such as ovarian cancer [12, 13, 22] and cervical cancer [23]. Thus, we established an in vitro model of NSCLC cell repopulation after lethal CDDP therapy. Using this in vitro model system, we studied whether adding MF following CDDP treatment is an efficacious strategy to abrogate repopulation of NSCLC cells leading to a better treatment outcome.

## Materials and methods

### Cell lines, culture conditions and treatments

Two NSCLC cell lines of different genetic backgrounds and sensitivities to platinum were selected for the study: A549 and H23. Both cell lines were obtained from the American Type Culture Collection (ATCC, Manassas, VA). A549 cells were isolated in 1972, from a 58 year-old Caucasian male who had bronchioalveolar lung cancer. It is considered a cell line of adenocarcinoma of type II alveolar cells that expresses wild type p53 and has low sensitivity to platinum [24]. H23 cells were derived from a non-small cell lung adenocarcinoma from a 51-year-old African American male. It is also considered a cell line of type II alveolar cells expressing mutant p53 yet with higher sensitivity to platinum agents when compared to A549 [24, 25]. Both lines were cultured in RPMI-1640 (Mediatech, Hendon, VA) supplemented with 10% fetal bovine serum (Atlanta Biologicals, Lawrenceville, GA), 20 mM HEPES (Mediatech), 4 mM l-glutamine (Mediatech), 0.45% D (+) glucose (Sigma Chemical Co., St. Louis, MO), 1 mM sodium pyruvate (Mediatech), 1× non-essential amino acids (Mediatech), 100 IU penicillin (Mediatech), 100 μg/ml streptomycin (Mediatech), and 0.01 mg/ml human insulin (Roche Diagnostics, Indianapolis, IN). Cells were maintained at 37 °C in a humidified atmosphere in the presence of 5% carbon dioxide.

Mifepristone (MF; Corcept Therapeutics, Menlo Park, CA) was dissolved in DMSO at a concentration of 20,000 μM and stored at − 20 °C. At time of treatment, the drug was thawed and introduced into media to reach final concentrations ranging from 5 to 40 μM. Cells were provided with MF-infused media chronically. Final concentration of DMSO in treatment groups ranged from 0.1 to 0.2%. Vehicle treated cells were provided with DMSO-infused media at appropriate maximum concentrations of the corresponding MF-treated cultures. Cisplatin (CDDP; Sigma) was stored in powder form until time of treatment. It was then dissolved in saline at a concentration of 3333 μM. The drug was introduced into the media to reach final concentrations ranging from 10 to 100 μM. Saline was provided to vehicle-treated cells. Cells received CDDP-infused media for 1 h, after which time media was removed, cells were washed with PBS, and media without CDDP was provided. The 1-h treatment time with CDDP was chosen because it mimics the amount of time CDDP is typically provided to a patient in a clinical setting. After the first group of dose–response experiments, the remaining groups of cells received for 1 h either 100 μM CDDP (for A549 cells) or 40 μM CDDP (for H23 cells). These concentrations were selected as they represent, respectively, 4 times the inhibitory concentration 50% (IC50) of CDDP for each cell line. These doses are supra-pharmacological but were selected to ensure that CDDP would cause maximal cytotoxicity to the cells, and to establish whether cellular repopulation could, nonetheless, occur. Clinically achievable concentrations of CDDP range between 6 and 10 μM [26–29]. In several experiments, CDDP/MF combinational treatments were given to the cells. In these cases, cells were first treated for 1 h with 100 μM CDDP (A549 cells) or 40 μM CDDP (H23 cells). Thereafter, CDDP-containing media was removed, cells were washed with PBS, and media containing either 10 or 20 μM MF was provided in a chronic manner. MF-infused media was refreshed every 48 h.

### Cell proliferation

Triplicate or sextuplet cultures of both cell lines were subjected to time-course experiments. Every 24 h, cultures were washed in PBS, trypsinized, pelleted by centrifugation at 500*g* for 5 min, and resuspended in PBS. Each sample volume was measured and 25 μl of each sample was combined with 225 μl of ViaCount reagent (Guava Technologies, Hayward, CA), resulting in a 1:10 dilution. The samples were then counted using the Guava ViaCount application in the Guava EasyCyte Mini microcapillary cytometer (Guava Technologies). The Guava ViaCount assay provides an absolute number of cell count by drawing cells into a capillary flow cell of known dimensions at a precisely controlled rate for specific amounts of time. The absolute cell counts depend on the dilution of the suspension, as well as of the total volume of sample from which the aliquot was taken. The data is both, acquired and analyzed, using the CytoSoft 4.1 software (Guava Technologies).

### Cell cycle analysis

Cells were washed in PBS, trypsinized, pelleted by centrifugation at 500*g* for 5 min, resuspended in PBS, fixed with 4% paraformaldehyde, and stored at 4 °C until further processing. Aliquots of approximately 150,000 cells were taken from each sample, washed in PBS, and centrifuged at 500*g* for 5 min. The supernatant was discarded and cellular aliquots were resuspended in 200 μl of cell cycle buffer [2.8 mM sodium citrate (Sigma), 7 U/ml RNAse A (Sigma), and 0.05 mg/ml propidium iodide (Sigma)] at a density of approximately 300 cells per μl. Cells were analyzed for their capacity to bind propidium iodide utilizing the Guava EasyCyte microcapillary cytometer. The cell cycle application of the CytoSoft 4.1 software (Guava Technologies) was used to analyze the results and to determine relative stages of the cell cycle.

### Phase contrast microscopy

Phase contrast microscopy was used to image non-treated cells, cells following exposure to treatments, and cells plated in clonogenic survival assays. Images were taken using a Zeiss Axiovert M200 inverted microscope (Carl Zeiss, Thornwood, NY). All images were taken with the objectives of 5× or 20×.

### Clonogenic survival assays

Five hundred viable cells from each treatment group were seeded in 6-well plates and cultured for 7 days until colonies were clearly discernable. At the end of the 7-day period, the medium was aspirated, the cells were washed with PBS, and then fixed with 100% methanol for 30 min. Thereafter, the cells were stained with a filtered solution of 0.5% (w/v) crystal violet (Sigma) for 10 min before being rinsed with tap water and dried at room temperature. Colonies of > 30 cells were scored manually using a Nikon Diaphot inverted microscope (Nikon, Garden City, NY). Clonogenic survival was expressed as the number of colonies formed under different treatment regimens.

### Statistical analysis

The concentrations of MF or CDDP that inhibited the growth of each cell line by 50% as compared with control cell growth (IC50) were calculated from data acquired in dose–response experiments using GraphPad Prism 5.0 (Graphpad Software, La Jolla, CA). The doubling time (DT) for each cell line was determined from growth curve experiments in which cell triplicates or sextuplets were plated at a density that allowed them to grow in culture for 96 h (A549 cells) or 120 h (H23 cells) without reaching confluence. Cells were harvested and counted by microcapillary cytometry as explained earlier. GraphPad Prism 5.0 (Graphpad Software) was used to conduct a non-linear regression analysis designed to estimate DT in culture. One-way analysis of variance (ANOVA) followed by Tukey’s Multiple Comparison post hoc test was used to compare the means of groups receiving different treatment regimens. Two-way ANOVA was used to determine interaction of dose and treatment over time. Specific post hoc tests employed are identified for each experiment. Differences were significant if p < 0.05.

## Results

### Exposure of cells to supra-pharmacological concentrations of CDDP for 1 h induces substantial toxicity, yet culture repopulation ensues with time

**Fig. 1:**
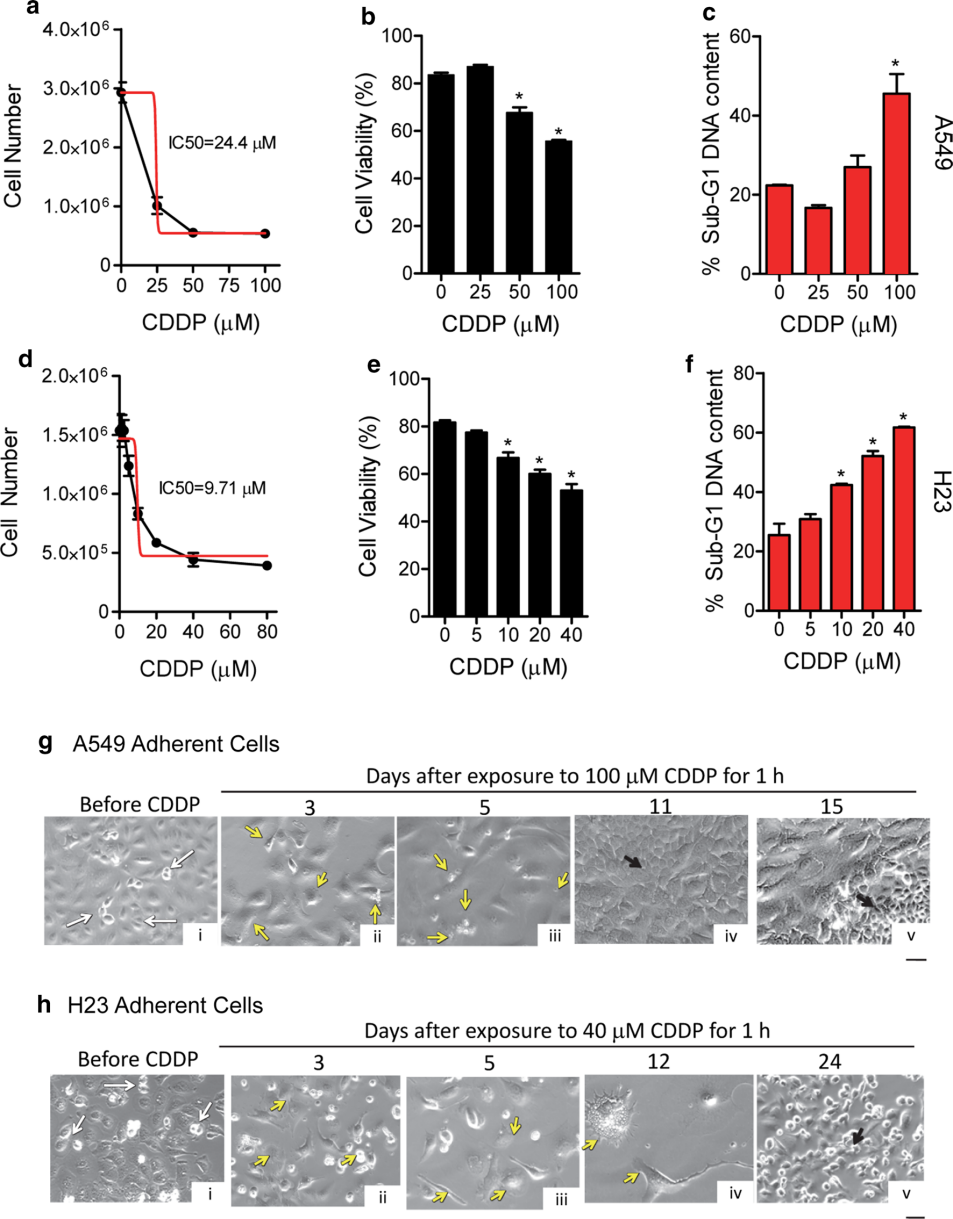
Sensitivity of NSCLC cells to CDDP and culture repopulation. A549 cells (**a**) or H23 cells (**d**) were seeded in six-well plates and given 2 days to attach before being treated with the indicated concentrations of CDDP for 1 h. Cells were then washed with PBS and provided untreated media. Five days later, cells were collected and counted using microcapillary cytometry (black line). The IC50 value was calculated using an algorithm from the GraphPad software (red line). Cell viability was assessed using the Guava ViaCount application (**b**, **e**), whereas hypodiploid DNA content was assessed by microcytometry upon propidium iodine binding (**c**, **f**). Phase contrast images were taken of adherent A549 (**g**) and H23 (**h**) cells, before and for various days after being exposed to CDDP for 1 h. White arrows indicate cells undergoing division. Yellow arrows depict signs of cellular damage. Black arrows depict clusters of cells undergoing active repopulation. Scale bar, 50 μm

Dose–response experiments were performed to determine the short-term response of the cells to CDDP. The sensitivity of the cells to the drug was quantified by determining the cell line’s IC50 value, or concentration of CDDP necessary to inhibit cell growth by 50%. The IC50 values were calculated 5 days following treatment as the largest toxicity was observed at this time point. A549 cells showed an IC50 value that was 2.5 higher than that of H23 cells (Fig. 1a, d) in coincidence with information found in the literature suggesting that H23 cells are more sensitive than A549 cells to CDDP [24, 25]. When viability was studied, 50% reduction was observed in A549 cells exposed to 100 μM CDDP whereas it took 40 μM CDDP to reduce viability by 50% in H23 cells (Fig. 1b, e). Accordingly, when we performed a cell cycle analysis of the samples to determine DNA content, the results support the viability data. Thus, in both cell lines, higher concentrations of CDDP resulted in increased number of particles with hypodiploid (a.k.a. Sub-G1) DNA content, consisting with apoptotic cell death.

A time-course experiment was conducted to study the long-term damage caused by supra-pharmacological doses of CDDP in terms of cellular morphology, and to assess whether or not, in prolonged times, cultures recovered as a consequence of repopulating cells. Results, shown in Fig. 1g, h display that, while before CDDP treatment there are signs of cellular division, such signs are lost 3–5 days after treatment for A549 cells or 3–12 days after treatment for H23 cells. Signs of damage are depicted by nuclear fragmentation, blebbing, cells with large cytoplasm, and giant cells. Of interest, by day 11 in A549 cells and day 24 for H23 cells following initial 1-h treatment with CDDP, the cultures show signs of cellular repopulation, suggesting that regardless of the supra-pharmacological concentration of CDDP used, there are cells escaping CDDP toxicity and repopulating the culture.

### Mifepristone inhibits growth of NSCLC cells regardless of their sensitivities to CDDP and in a dose- and time-dependent manner

**Fig. 2:**
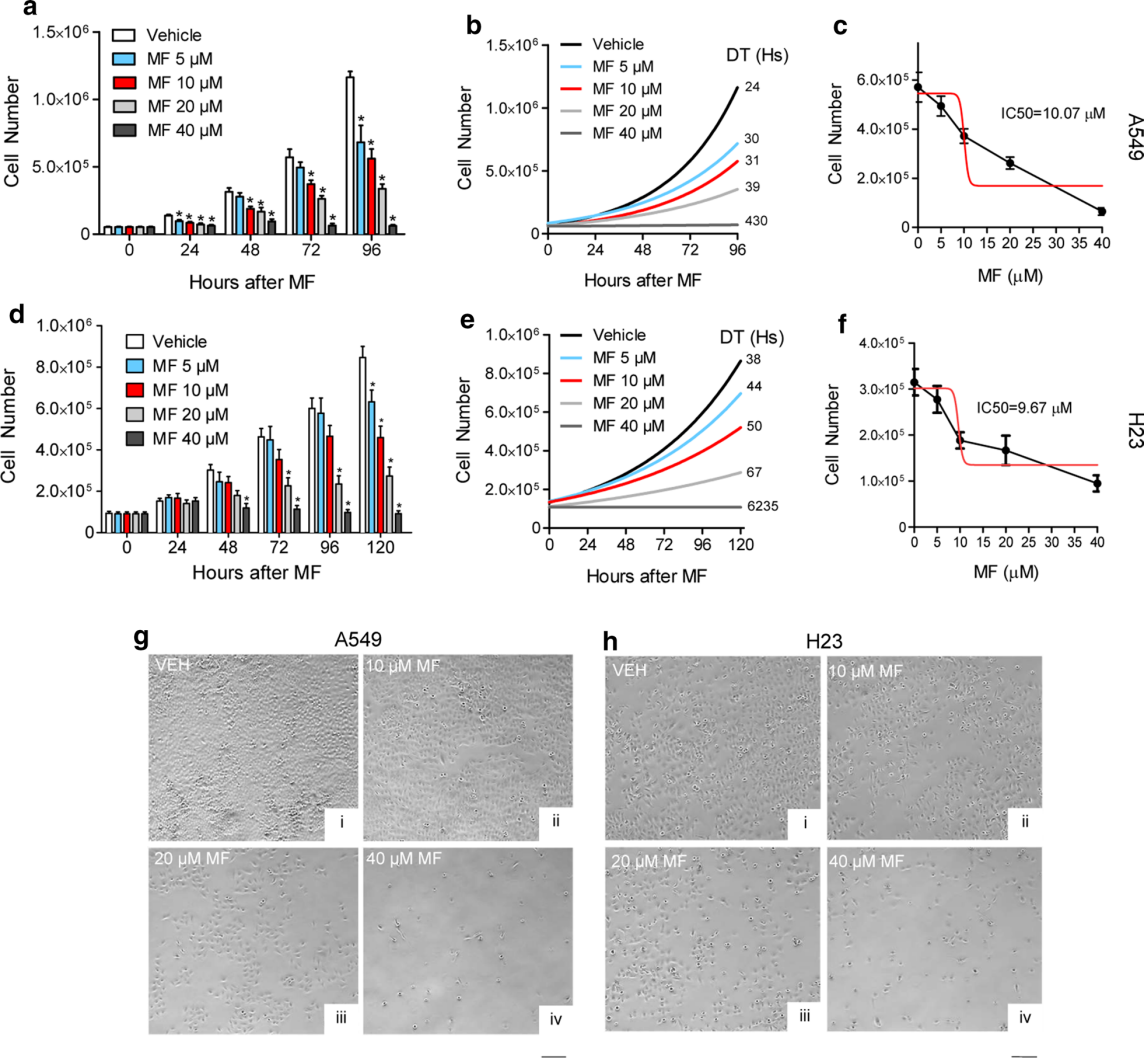
Effect of MF on the growth of NSCLC cells. A549 cells (**a**) or H23 cells (**d**) were seeded in six-well plates, treated with the indicated concentrations of MF, and counted at regular intervals. Results were analyzed using two-way ANOVA followed by Dunnett’s post hoc test. *Indicates p < 0.05 compared to vehicle at equivalent time point. In **b** and **e**, the doubling time (DT) for cells under each treatment regime was computed using a non-lineal exponential growth algorithm and is indicated next to each line. In **c** and **f**, A549 cells and H23 cells, respectively, were seeded and allowed to attach for 24 h before treatment with the indicated concentrations of MF. Following 72 h, cells were collected and counted using microcapillary cytometry (black line) and the IC50 calculated (red line). Panels **g** and **h** depict phase contrast microscopy images of the cultures following 3 days of MF treatment. Scale bar, 200 μm

To determine the effect of MF on the growth of NSCLC cells, three independent dose–response and time-course experiments were performed for both cell lines. As early as 24 h, it was evident that MF inhibited the growth of the cells, even at the lowest concentration of 5 μM. It was also evident that the growth inhibition had a dose-dependent fashion. Performing a two-way ANOVA supported this observation by showing an interaction of treatment and time with a p value lower than 0.01. In addition, Dunnett’s post hoc test showed significant differences between vehicle- and MF-treated cells at most of the time points and concentrations studied (Fig. 2a, d). The decreased rate of proliferation in the presence of MF was reflected by the longer doubling times of the cells subjected to the various concentrations of MF. In both cell lines, doubling times consistently increased with increased concentration of MF (Fig. 2b, e). Notice that the extremely large doubling times for cells treated with the largest dose of MF (40 μM), represents a theoretical number as the cells no longer proliferate—they actually die—when subjected to this high concentration of the antiprogestin/antiglucocorticoid. Of interest, when the amount of MF needed to block the growth by 50% (i.e. IC50) was calculated, both A549 and H23 cells showed a similar IC50 value of ~ 10 μM (Fig. 2c, f). This is relevant in lieu of the fact that both cell lines had a very different sensitivity to CDDP (Fig. 1a, d). The dose-dependent decrease in number of cells upon MF action can also be visualized via microscopy. Images, shown 72 h after exposure to MF, display clear dose-related decreases in cell number (Fig. 2g, h).

### Mifepristone-induced cytostasis associates with profound morphological changes, yet it is a reversible phenomenon

**Fig. 3:**
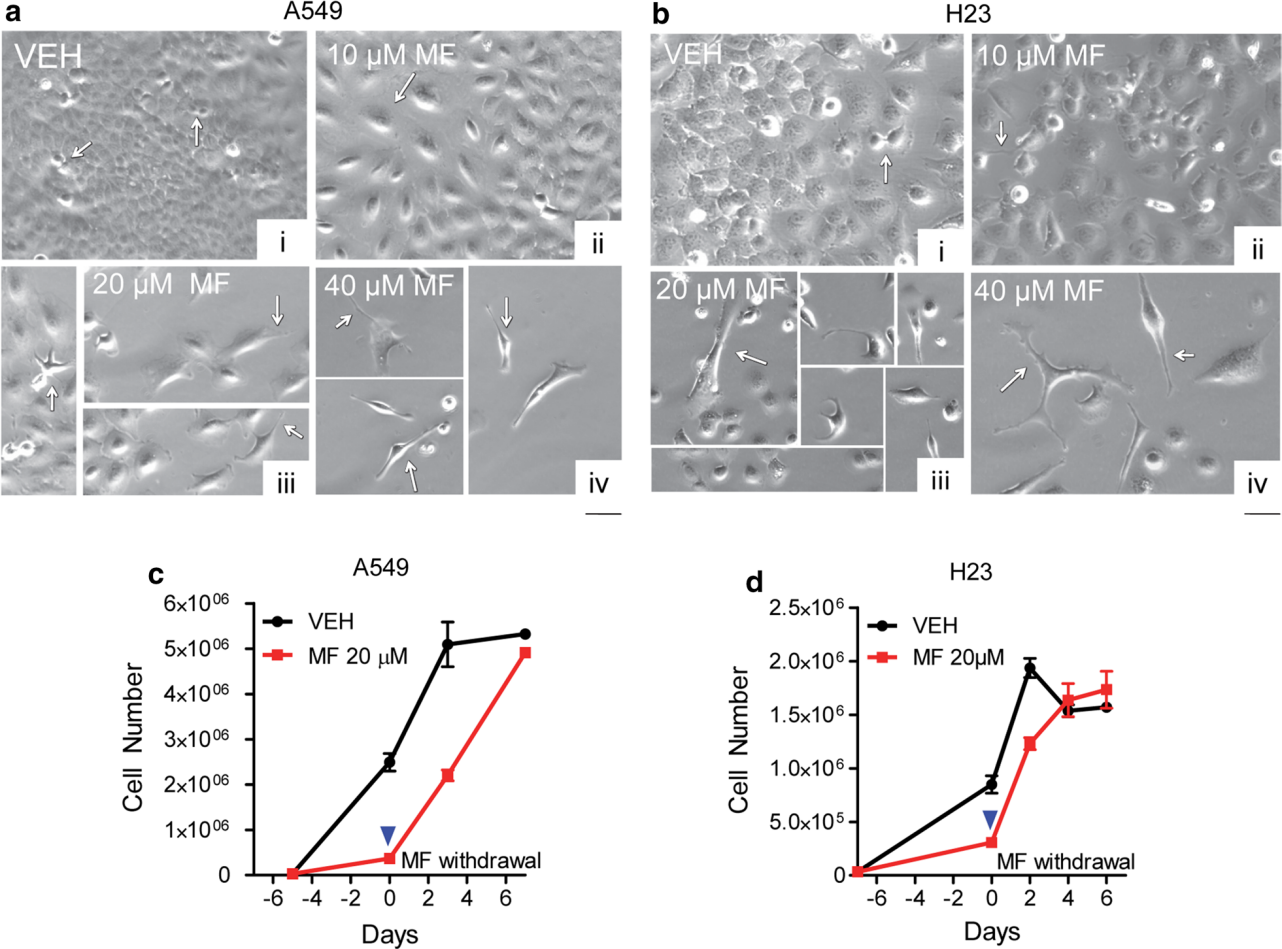
Morphological features of NSCLC cells following MF exposure and the consequence of MF withdrawal. In **a** and **b**, A549 cells and H23 cells, respectively, were seeded and allowed to attach for 24 h before being treated with the indicated concentrations of MF. Images were taken using phase contrast microscopy following 3 days of treatment. Vehicle-treated cells continued to proliferate (arrows in [i]). Changes in MF-treated cultures included increased cellular size (arrows in [ii]), branching and cytoplasmic extensions with spindle-like morphology (arrows in [iii and iv]. In **c** and **d**, A549 cells and H23 cells, respectively, were released of MF treatment on day 0 (blue arrowhead) by replacing MF-containing media with media devoid of MF. At regular intervals, cells were counted using microcapillary cytometry. Negative days are days during which cells were exposed to MF

There were clear morphological changes in the cultures of NSCLC cells following treatment with MF. In A549 cells and after 96 h of treatment, morphological changes were varied, including increased size of cytoplasm, and cytoplasm branching with pronounced extensions, giving the cells a spindle-like morphology. The proportion of spindle-like cells in culture increased as the doses of MF increased (Fig. 3a). H23 cells, upon incubation with various doses of MF for 120 h, also showed extensive morphological changes; the most striking of these changes was again the extensive cellular branching (Fig. 3a). Cells from this experiment were subjected to a cell viability assay. It was observed that MF was cytostatic up to the concentration of 20 μM. However, 40 μM of the drug reduced the viability of both cell lines to approximately 70%, which was associated with an increase in hypodiploid DNA content to about 30% (data not shown), indicating that the reduced number of cells observed under 40 μM treatment is consequence not only of reduced cell division but also due to cell death.

To further study whether NSCLC cells can re-establish proliferation following withdrawal of MF, cells were seeded, allowed to attach, and treated with 20 μM MF for 4 days (A549 cells) or 5 days (H23 cells). MF-containing media was then aspirated and replaced with MF-free media. Every 2 days and for a total of 6 days of further incubation, cells were trypsinized and counted using microcapillary cytometry. Both A549 and H23 cells were able to re-establish growth when provided with normal media free of MF, with a kinetics of proliferation that seems faster than that of untreated cells (Fig. 3c, d), while reverting their morphology as well (data not shown). The apparent faster recovery of MF-pretreated cells could be consequence of cell cycle synchronization and exponential growth allowing MF-pretreated cells to reach levels of proliferation, 4 days after drug withdrawal, similar to that of untreated cells.

### Intertwining cytostatic doses of mifepristone in between CDDP-free treatment intervals prevents repopulation of cells escaping the lethality of CDDP

**Fig. 4:**
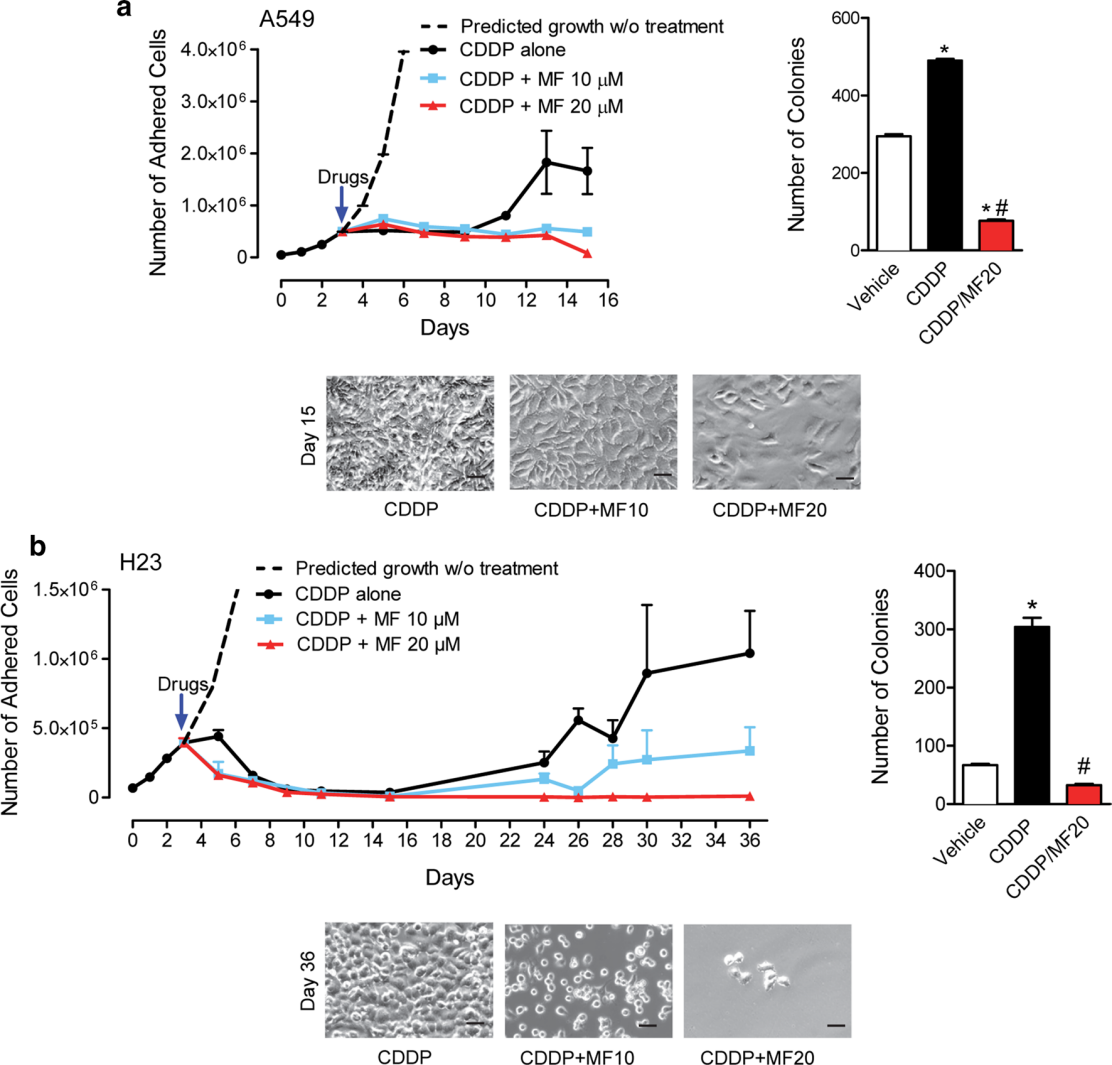
Blockage of repopulation of NSCLC cells by MF following CDDP exposure. In **a** and **b**, A549 cells and H23, respectively were seeded in six-well plates and allowed to establish exponential growth. On day 3, cells were treated with either CDDP for 1 h or CDDP for 1 h directly followed by chronic treatment with 10 μM MF or 20 μM MF for the indicated times. Cell number was counted at 2-day intervals using microcapillary cytometry. The right side of the panel shows the clonogenic survival assessed by plating 500 viable cells taken from day 15 cultures (A549 cells) or day 36 cultures (H23 cells) in sextuplets and provided normal media for 7 days. At the end of the incubation, cells were fixed with methanol, and stained with crystal violet. Colonies with > 30 cells were counted as positive colonies. Results were analyzed using one-way ANOVA followed by Tukey’s Multiple Comparison Test. *Indicates p < 0.05 compared against vehicle; ^#^indicates p < 0.05 compared against CDDP. For A549 cells, CDDP was used at a 100 μM concentration. For H23, CDDP was used at a 40 μM concentration. The lower side of the panels depicts phase contrast images of the treated cultures on day 15 (A549 cells) or 36 (H23 cells)

After assessing the independent effects of CDDP and MF, the effect of chronically providing MF following initial CDDP treatment was evaluated. Long-term experiments for each cell line created an in vitro model of tumor cell repopulation intended to re-enact the clinical recurrence observed in patients. To accomplish this, cells were plated in triplicates and allowed to establish exponential growth. They were then treated for 1 h with a supra-pharmacological concentration of CDDP, equal to four times the IC50 value for each cell line. For A549 cells, this dose was 100 μM; for H23 cells, it was 40 μM. After CDDP treatment, media was removed, cells were washed with PBS, and new media was provided containing either vehicle (DMSO), 10 μM MF, or 20 μM MF. DMSO and MF-containing media were refreshed every 2 days. In both cell lines, despite the high doses of CDDP given, cells were capable of repopulating over time. In A549 cells, this repopulation began 8 days following treatment, whereas in H23 cells, repopulation began 20 days after treatment. Chronic exposure of the cells to MF following CDDP inhibited such repopulation in both cell lines studied and in a dose-dependent manner (upper left panels in Fig. 4a, b). It seems that after 15 days of CDDP treatment in A549 cells, and 36 days of CDDP treatment in H23 cells, the chronic presence of 20 μM MF completely abrogated cellular repopulation, yet without totally eliminating cells from the culture (lower panels in Fig. 4a, b). These results were confirmed in clonogenic survival studies. A549 or H23 cells were taken from day 15 or day 36 cultures, respectively from previous experiments (left panels, Fig. 4a, b). The clonogenic capacity of CDDP-exposed cells was higher than that of cells never receiving treatment, suggesting accelerated repopulation; such repopulation was abrogated by the chronic presence of MF (right panels, Fig. 4a, b).

### Mifepristone greatly improves second-line treatment with CDDP

**Fig. 5:**
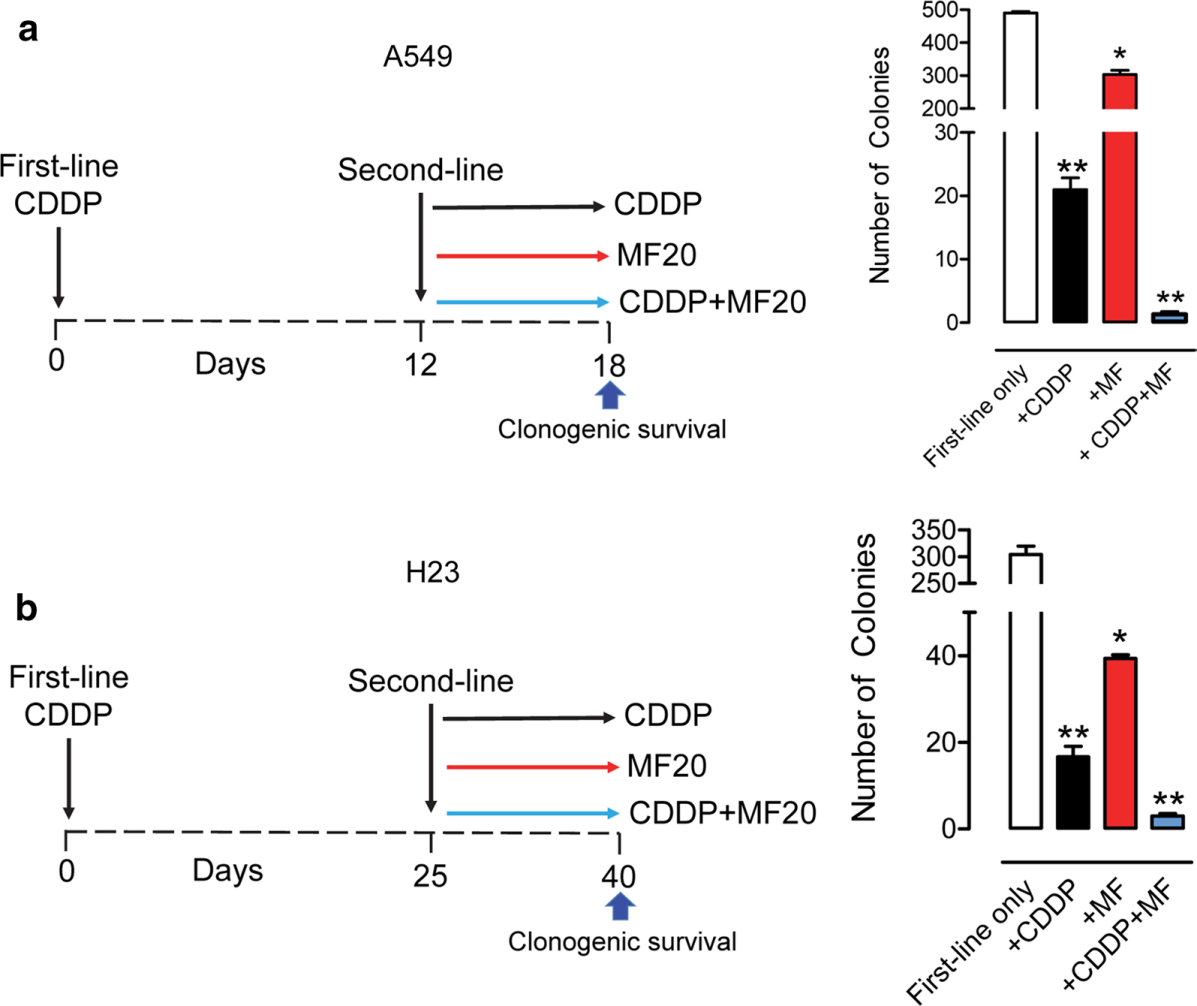
Clonogenic capacity of NSCLC cells following second-line chemotherapy. **a**, **b** The treatment schedule for A549 and H23 cells, respectively. Twelve days after first-line treatment (A459 cells) or 25 days after first-line treatment (H23 cells), a second-line treatment was given. At the end of the experiment (day 18 for A549 cells or day 40 for H23 cells), 500 viable cells taken upon second-line treatment with vehicle (indicated as ‘first-line only’ in right panels), CDDP, 20 μM MF, or CDDP plus 20 μM MF were seeded and provided untreated media to assess their clonogenic survival. Cells were allowed to proliferate for 7 days, at which time media was aspirated, cells were washed with PBS, fixed with methanol and stained with crystal violate. Colonies having > 30 cells were considered positive and consequently manually counted. Results were analyzed using one-way ANOVA followed by Tukey’s Multiple Comparison Test. *Indicates p < 0.05 and **indicates p < 0.01 compared against ‘first-line only’ groups. For A549 cells, CDDP was used at a 100 μM concentration. For H23, CDDP was used at a 40 μM concentration

Despite the high concentrations of CDDP given to the cells for 1 h, over time, they always repopulated unless exposed chronically to MF. To study what intervention would be more efficient for cells escaping first-line CDDP, a second-line treatment with CDDP, MF, or the combination of CDDP/MF was given on repopulation day 12 (for A549 cells) or repopulation day 25 (for H23 cells), and a clonogenic survival assay was performed after a total of 18 days of culture for A549 cells, and of 40 days for H23 cells (left panels in Fig. 5a, b). Data shown in the right panels of Fig. 5a and b depicts that the clonogenic survival of repopulating cells after first line CDDP treatment was significantly reduced by a second dose of CDDP for 1 h, or by the continuous presence of MF. However, when the treatments were combined, i.e. when second-line exposure to CDDP for 1 h was followed by chronic exposure to 20 μM MF, the clonogenic survival was reduced to a negligible number of colonies.

## Discussion

We created an in vitro model of NSCLC growth, response to chemotherapy, and cellular repopulation. This model intends to mimic the behaviour of NSCLC recurrence; NSCLC develops, enters remission following treatment, and recurs over time. We focused on enhancing the gold standard treatment of advanced NSCLC: platinum-based therapy. We purposefully selected two NSCLC cell lines of disparate sensitivities to CDDP and tested the hypothesis that chronic MF exposure would independently inhibit cell growth and, in combination with CDDP, abrogate repopulation of cells that escape cytotoxic CDDP treatment. We utilized the 1-h exposure to CDDP paradigm and a supra-pharmacological concentration to maximize lethality while maintaining a clinically-relevant time of exposure. Although the majority of the cells in culture died because of such treatment approach, there were isolated cells that survived the treatment. Such cells, because of their scarcity, needed extended time in culture to resume proliferation. We were able to document repopulation of NSCLC cells upon exposure to fourfold their CDDP IC50’s. The results highly suggest that repopulating cells somewhat escaped the lethality of CDDP. In our study, A549 cells and H23 cells repopulating after CDDP exposure show an apparent increased clonogenic capacity when compared to exponentially growing, vehicle-treated cells. These data suggest that in our model system there is either a synchronization of the cells that remain in culture following CDDP exposure, which could explain the higher number of positive colonies formed at the same time, or instead, the increased clonogenic survival could be a product of accelerated repopulation of CDDP-exposed cells.

When given on its own, MF treatment inhibited growth of both A549 and H23 cells. The translational relevance of this result, is that if the disease is detected early enough, a patient could be provided with a daily MF treatment that would inhibit growth of, and complications from, NSCLC. Supporting this possibility, it is known that MF is well tolerated in humans [30]. Although the cancer would still be present under the cytostatic pressure of MF, it would be contained as a manageable chronic disease. This is supported by data generated from two anecdotal patients with advanced metastatic NSCLC, in which long-term high-quality survival was achieved using oral MF [31]. These promising results led to an ongoing single-stage phase II study of MF in patients with advanced or metastatic NSCLC who have failed two or more previous chemotherapy regimens (https://clinicaltrials.gov/ct2/show/NCT02642939).

When working as a cytostatic agent, MF, used as monotherapy, caused a remarkably change in the morphology of the cells which displayed spindle-like extensions. Our laboratory previously demonstrated, using ovarian, breast, glioblastoma, and prostate cancer cells, that such morphological changes caused by MF are associated to reduced adherence [32]; this reduced adhesive capacity was consequence of membrane ruffling, which involves a disproportionate redistribution of fibrillar actin to ruffles that are sheet-like membrane protrusions of flat membrane folds from the cortical cytoskeleton that do not attach to extracellular matrix [33].

However, exciting the possibility of using MF as monotherapy is, we believe that the most potent use of MF is in combination with a lethal dose of CDDP. When we explored the effect of MF on NSCLC cells after treatment with CDDP, we observed that CDDP could significantly damage cells and decrease cell numbers, but that some cells would escape treatment and eventually repopulate over time. It is likely that in the clinic these cells regenerate the tumor following treatment [17]. We found that MF completely abrogated the repopulation of such ‘escape cells’ following CDDP treatment. The clinical translation of this finding could be enormous; MF could provide an avenue to prevent recurrence of NSCLC, a phenomenon that commonly results in patient’s death.

A cytostatic therapy against cancer cell repopulation following lethal chemotherapy such as the one provided by MF, has been proven for other compounds. For example, selective estrogen receptor modulators were shown to block repopulation of breast cancer cells exposed to 5-fluorouracyl and methotrexate in vitro and in vivo [34, 35]. Furthermore, in prostate cancer, a mammalian target of rapamycin inhibitor delayed the growth of tumor xenografts in immunosuppressed mice if given following mitoxantrone and paclitaxel [36].

Although the link between repopulation of escape cells and recurrence in the clinic seems to be evident, how this occurs is unknown. There is evidence showing that cancer cells escape CDDP-induced DNA damage by undergoing reverse ploidy, also known as neosis [37–40]. Polyploid giant cell formation is a characteristic development following CDDP treatment [13, 40]. For decades, these cells were considered reproductively dead. Yet, researchers have shown that they have facility, in certain percentage, to give rise to diploid or near diploid (paradiploid) cells capable of proliferation [37–41]. When these giant multinucleated cells were separated from cells of normal DNA content, it was shown that only the prior eventually repopulated following irradiation [38].

In our study, following toxic CDDP treatments, A549 and H23 cells showed extensive signs of damage, including the formation of very large cells. Yet, nascent colonies containing growing small cells were observed in both cell lines after being exposed to CDDP. In contrast, in cultures that received MF treatment in addition to CDDP, an overall reduced number of cells remained in the plates. These cells persistently showed a predominantly giant phenotype and a smaller population of cells never became re-established. Previous research in our laboratory demonstrated that ovarian cancer cells also display a similar giant phenotype population following CDDP treatment. We have shown that these cells eventually die in culture as demonstrated by marked cleaved PARP positivity in CDDP/MF treated cells, but not in those receiving CDDP monotherapy [13]. Similar mechanism likely occurred in cancer cells repopulating after CDDP/paclitaxel combination therapy; in such study we observed a population of cells with hyperploid DNA content that was reduced in parallel to cell repopulation; such hyperploid cell population, however, disappeared after MF exposure in favor of hypodiploid DNA content, suggesting that cells receiving MF after chemotherapy die instead of returning to the cell cycle [12].

Furthermore in ovarian cancer cells, polyploid giant cells purified from other cancer cells under the stress of hypoxia, generated live, regular-sized cancer cells with stem-like cell properties very rapidly via budding and bursting [42]. This suggest that MF could inhibit the ability of giant polyploid cells to give rise to diploid or paradiploid cells of normal replicative capacity, and/or cause these cells to die. Ploidy reversal, observed in vivo and in vitro [43], is a biological phenomenon not only occurring in cells in response to cytotoxic agents [44], but also during organ development [45] and tissue regeneration [46]. Cumulatively, this data suggest that MF could inhibit repopulation of NSCLC cells escaping treatment with CDDP by blocking reverse ploidy.

Nonetheless, MF therapy could also abrogate repopulation of cancer cells by targeting a range of other survival mechanisms. For instance, it was recently demonstrated that irradiated dying cancer cells secrete prostaglandins in response to caspase 3-mediated activation of the arachidonic acid metabolic pathway, leading to the stimulation of surviving cells to proliferate [47, 48]. Perhaps, MF works to block these signals. MF could also inhibit the growth of a small subpopulation of tumor initiating cells that are resistant to CDDP-based therapy. Supporting this theory, a genetic evolution study of high-grade serous ovarian adenocarcinomas suggested that pre-existing minor clones may remain following CDDP treatment, and that their proliferation could actually be enhanced by the treatment [49]. According to this scenario, MF would block the repopulation of cells that never responded to CDDP. This explanation is supported in that almost identical amounts of MF were required to elicit antigrowth effects against both NSCLC cell lines despite their drastic differences in sensitivity to CDDP. Our results clearly demonstrate in vitro that MF is an attractive supplement to CDDP therapy regardless of tumor sensitivity to CDDP. Moreover, if MF is not provided as part of first-line treatment, our data suggest that it is also efficacious as a second-line option. In both NSCLC cell lines, when given in combination with CDDP as a second-line measure, MF almost obliterated the clonogenic capacity of the treated cells. Thus, MF could serve to increase the efficacy of second-line treatment without increasing toxicity.

## Conclusion

We demonstrated that MF caused growth inhibition in two NSCLC cell lines independently of their sensitivities to CDDP or p53 background. These results support the use of MF to inhibit cell growth during early stages of NSCLCs. Following treatment with CDDP, MF abrogates repopulation of cells escaping CDDP therapy. This finding supports using chronic, low toxic MF therapy as adjuvant for standard CDDP treatment in advanced NSCLCs, which is particularly enlightening considering that long-term (months to years) of daily administration of MF is feasible and clinically well tolerated [50].

## Abbreviations

DMSO: dimethyl sulfoxide
ANOVA: analysis of variance
HEPES: 4-(2-Hydroxyethyl)piperazine-1-ethanesulfonic acid, *N*-(2-Hydroxyethyl)piperazine-*N*′-(2-ethanesulfonic acid)
MF: mifepristone
CDDP: cisplatin
NSCLC: non-small cell lung carcinoma
